# The Impact of Vavilov’s Concept of the Centres of Crop Origin and Diversity on Research, Conservation, and Utilisation of Plant Genetic Resources Today: A Review on the Occasion of Vavilov’s 135th Anniversary

**DOI:** 10.3390/plants12142685

**Published:** 2023-07-19

**Authors:** Igor G. Loskutov, Andreas W. Ebert, Axel Diederichsen

**Affiliations:** 1 N.I. Vavilov All-Russian Institute of Plant Genetic Resources (VIR), 42-44 Bolshaya Morskaya Street, 190000 St. Petersburg, Russia; 2 World Vegetable Center, D-73529 Schwaebisch Gmuend, Germany; 3 Plant Gene Resources of Canada, Saskatoon Research and Development Centre, Agriculture and Agri-Food Canada, Saskatoon, SK S7N 0X2, Canada

This Special Issue of *Plants* is dedicated to the eminent scientist Nikolai Ivanovich Vavilov (1887–1943) in remembrance of his 135th birthday on 25 November 1887. He was, without doubt, one of the greatest protagonists of the “plant genetic resources movement” [1]. Vavilov’s work has had a substantial impact on the global community of researchers, conservationists, and even political decision-makers worldwide, especially those with an interest in the fields of botany, genetics, and plant breeding; the evolution of cultivated plants; and the conservation and utilisation of plant genetic resources.

Vavilov grew up in a well-off merchant family in Moscow, a fact that did not earn him credit from the communist dogmatists. Despite his urban upbringing, he decided to pursue a career in agricultural science. He graduated from the Agricultural Academy at Petrovsko-Razumovsky (Moscow) and continued his studies and early research work at Cambridge University in England [2]. He was a student of William Bateson, the pioneering geneticist of the day, and worked as a researcher under him at the John Innes Horticultural Institute, which Bateson had founded. While based in England, he started to investigate the genetic basis of the resistance of cereals to fungal diseases in Mendelian terms and described and published his findings [3]. At the start of the First World War, Vavilov returned to Russia and developed his theory on the importance of geographical origin on the genetic diversity of plants. He elaborated on the geographical interrelationships between the genetically determined traits of the diversity within a cultivated plant’s species and integrated the concepts of A. de Candolle and C. Darwin into his theory. He stood in the tradition of great explorers and botanists such as C. Linnaeus, C.L. Willdenow, and A. von Humboldt but focused mainly on cultivated plants and their wild progenitors. He was among the first to claim that the diversity of cultivated plants and their wild relatives can contribute to the understanding of the history of human civilisation and that such studies deserve similar attention as archaeological artefact studies undertaken by historians. Vavilov also considered linguistic aspects when trying to understand the geographical movement of cultivated plants.

In addition to his fundamental theoretical works on the centres of crop origin and diversity, he emphasised the need for and importance of collecting and conserving cultivated plants and their wild relatives ex situ to make them available for research and breeding programs and use by future generations. All of this has formed a solid scientific basis for ongoing efforts to conserve plant genetic resources in genebanks, both nationally and internationally.

Vavilov’s major scientific contributions are largely reflected in his publications: “The law of homologous series in variation” [4], “Centers of origin of cultivated plants” [5], and “Linnaeus species as a system” [6]. The main regularity determining the diversity of a Linnaean species has been referred to by Vavilov as the law of homologous series in (hereditary) variation within the boundaries of the same genus [6]. As an outstanding universalist, Vavilov supported an integrative, interdisciplinary approach to science and strived to develop a holistic view of the evolution of cultivated plants. In addition to the biological sciences, geography was the discipline Vavilov saw as most relevant. He advocated a differential systematic and a differential geographic approach supported by morphological, physiological, cytological, and hybridological methods to assess intraspecific diversity and determine cultivated plants’ centres of origin. He continued to revise his views on the origin of cultivated plants, and several publications up to 1940 show how new insights changed his original ideas published since 1919. His concepts of the law of homological series in the variation of plant species, the centres of origin and taxonomy of cultivated plants and their wild relatives, genetic diversity, and crop evolution have inspired generations of researchers working on this subject [7,8]. Vavilov and his associates explored the five continents of the world and collected unique agrobiodiversity in very remote regions [9], a lot of which is being preserved by the N.I. Vavilov Institute for Plant Genetic Resources in St. Petersburg, the Russian Federation. His germplasm collections have been researched and utilised in plant breeding projects worldwide. Remarkably, Vavilov’s final work is said to have been a monograph on the history of the world’s agriculture [3], published in the collection of selected works [9]. This clearly shows that, despite all specialised studies, Vavilov never lost sight of the important cultural role of agriculture for humankind.

Vavilov was convinced that science would always prevail and did not fear his detractors’ malicious statements. One such detractor was T.D. Lysenko, who started to promote views on heredity that were more appealing to immaterial dogma and to fight against the Vavilov school of thought, together with like-minded scientists and officials who made administrative decisions. Sadly, in 2023, it will be 80 years since Vavilov died in 1943 in a prison in Saratov in the Soviet Union as a victim of Stalinism.

We are pleased that experts in the field of plant genetic resources for food and agriculture from various disciplines volunteered time and resources to contribute to this Special Issue of the journal *Plants* to show how the field has evolved since Vavilov’s fundamental contributions.

This Special Issue comprises seven published papers covering a wide array of aspects, from collecting information on world collections of citrus crops to studying native potato varieties collected by Vavilov during his expeditions. Other contributions focus on disease resistance of rare wheat species maintained in the VIR collection, concerning Vavilov’s concepts of plant immunity; the diversity of cultivated oat species of the VIR collection in terms of nutritional value; phenotypic characterisation and diversity analysis of the root system in soybeans in the Korean gene bank; the global collection of crop wild relatives in different parts of the world; and, finally, the influence of Vavilov’s ideas and activities on the formation of plant genetic resource collections and the creation of a gene bank in Scandinavia.

During his expeditions, Vavilov observed the genetic diversity of citrus species and recognised their economic potential. In an article on the global conservation of citrus diversity Volk et al. (2023) conducted an online survey and analyzed data from 43 collections in 27 countries. The six largest citrus collections have 1000 to 1735 accessions; mandarins and sweet oranges are represented with the greatest number of accessions. Other citrus crops maintained in genebanks around the globe include lemon, pummelo, grapefruit, hybrids, lime, sour orange, citron, kumquat, papeda, finger lime, and crop wild relatives. Due to strict quarantine regulations, diseases pose significant threats to citrus collections and often impede germplasm exchange. Thanks to intensive sanitation efforts, a few collections are currently maintained in a clean-plant state. Investments in financial and human resources are necessary to ensure the long-term safety and security of global citrus collections [10].

Samples from the WIR herbarium at VIR and living samples from the VIR field genebank, collected after the appearance of *Phytophthora infestans* in Chile, were used by Gavrilenko et al. for a comparative analysis of the genetic diversity of Chilean cultivated potatoes [11]. Using a set of 20 plastid DNA-specific markers, two plastid DNA types, T and A, and two chlorotypes were identified in herbarium specimens, with a clear predominance (96%) of chlorotype cpT_III. The living accessions were differentiated into four chlorotypes. The discovery of a D-type cytoplasm in living Chilean accessions that possess two new chlorotypes indicates a replacement of native cultivars and introgression from the wild Mexican species *S. demissum*. This wild species was actively used in potato breeding as a source of race-specific resistance to late blight.

To identify new sources of effective resistance to four foliar diseases of wheat (leaf rust (*Pt* = *Puccinia triticina*), powdery mildew (*Bgt* = *Blumeria graminis tritici*), *Septoria nodorum* blotch (SNB), and dark-brown leaf spot blotch (HLB = Helminthosporium leaf blotch), 173 accessions of four wheat species, *Triticum boeoticum*, *T. urartu*, *T. araraticum*, and *T. dicoccoides*, from the VIR collection were tested at the juvenile and adult growth stages [12]. Two samples of *T. boeoticum*, three of *T. urartu*, and one of *T. araraticum* were resistant to leaf rust at both tested stages. High levels of *Bgt* resistance were identified in three entries of *T. boeoticum*, one of *T. araraticum*, and eleven of *T. dicoccoides*. All tested accessions were susceptible to HLB and SNB at both developmental stages. Accessions identified as resistant to specific diseases represent valuable plant material for introgressive hybridisation in bread and durum wheat breeding. The results are discussed in the context of N.I. Vavilov’s concept of crop origin and diversity and the laws of plant natural immunity to infectious diseases.

Popov et al. evaluated the nutritional and agronomic value of 49 accessions of cultivated oat species maintained in the VIR genebank [13]. Sources of useful agronomic traits were identified, which can be used to develop new high-yielding and highly nutritious oat cultivars adapted to local cultivation conditions. The authors demonstrated that the VIR collection has great potential for breeding new oat cultivars for human and animal consumption. Kim et al. studied the root phenotype of 374 soybean accessions maintained at the RDA genebank in South Korea [14]. For this purpose, the authors used a high-throughput method and assessed eight root morphological traits. IT208321, IT216313, and IT216137 were identified as elite soybean accessions for root traits and are, therefore, candidates for breeding programs.

Eastwood et al. report that from 2013 to 2018, partners from 25 countries undertook expeditions on five continents to collect seeds of crop wild relatives of 28 crops of global agricultural importance for climate change adaptation [15]. More than 4500 unique seed samples from at least 355 CWR taxa were collected, conserved *ex situ*, and safety duplicated in national and international genebanks. These accessions are available for distribution under the Standard Material Transfer Agreement of the International Treaty. Several newly collected samples have already been used in pre-breeding programs to adapt crops to future challenges.

In the review of Solberg et al. [16], the authors examined Vavilov’s relationship to Scandinavia and the impact he and his ideas have had on the conservation and use of crop germplasm in Scandinavia. Vavilov’s interaction with scientists in Scandinavia established the Nordic Gene Bank (today: NordGen) in 1979 in Sweden and, later, the Svalbard Global Seed Vault in Norway, which started operations in 2008. Vavilov did not recognise Scandinavia as part of a centre of origin of cultivated plants. However, at a later stage, P.M. Zhukovsky developed the concept of mega-centres of the diversity of cultivated plants. Scandinavia became part of what he termed the European–Siberian Region of Diversity. The authors list the species domesticated in Scandinavia or Northern Europe, discuss concepts related to crop evolution, and highlight the significant impact Vavilov has had by inspiring scientists across disciplines and over many decades.

The publication of this Special Issue, dedicated to the birth anniversary of N.I. Vavilov, shows that the ideas and works of this great scientist of the 20th century are still relevant and will remain in demand in the 21st century and beyond.
